# Surgical Repair of Popliteal Artery Aneurysms Still Represent the Gold Standard: A Contemporary Cohort Study from a High-Volume Centre and Comparison with Contemporary Endovascular Series

**DOI:** 10.3390/diagnostics15202608

**Published:** 2025-10-16

**Authors:** Ottavia Borghese, Teresa Lodico, Simone Cuozzo, Yamume Tshomba

**Affiliations:** 1 Unit of Vascular Surgery, Fondazione Policlinico Universitario A. Gemelli I.R.C.C.S, 00168 Rome, Italy; ottaviaborghese@gmail.com (O.B.); teresa.lodico01@icatt.it (T.L.); simone.cuozzo@guest.policlinicogemelli.it (S.C.); 2 Department of Cardiovascular Sciences, Vascular and Endovascular Surgery School, Faculty of Medicine and Surgery, Catholic University “Sacro Cuore”, 00168 Rome, Italy

**Keywords:** aneurysm, open repair, surgical bypass, endovascular procedure, popliteal artery, limb ischemia

## Abstract

**Background/Objectives:** Despite their low incidence, popliteal artery aneurysms (PAAs) are the most common aneurysms of the peripheral arteries and carry a high risk of limb loss. The optimal treatment, either with open (OR) or endovascular repair (ER), remains debated due to the lack of high-level evidence. **Methods:** In accordance with STROBE guidelines, we conducted a retrospective observational study with a prospective follow-up. All patients presenting with a PAA who underwent elective OR in the Vascular and Endovascular Surgery Unit of Agostino Gemelli Hospital (Rome, Italy) over the last four years were enrollved. Urgent and endovascular cases were excluded. Clinical examination, Doppler ultrasound (DUS), and contrast-enhanced computed tomography angiography (CTA) were performed preoperatively. Clinical and DUS follow-up was performed at 1, 3, 6, and 12 months postoperatively and annually thereafter. Endpoints were the primary, primary assisted, and secondary patency calculated using a Kaplan–Maier estimate based on the “first event” (arterial stenosis, occlusion, or reintervention) after the procedure. Amputation rate and overall mortality were also assessed. The results were compared with the success and complication rates reported in contemporary endovascular series. **Results:** Overall, 62 open interventions for popliteal artery aneurysms were performed during the study period; 49 patients (100% male, 70.3 SD ± 8.8 years) were included for a total of 52 PAAs treated electively (median diameter 30.5 mm, range 20–75; 92.3% fusiform). Aneurysm involved P1 segment in 38.5% of cases (20), P2 in 48.1% of cases (25), and P3 in 13.5% of cases (7). Two runoff vessels were present in most patients (37, 71.2%). Surgery consisted of the aneurysm’s exclusion through ligation and autologous vein or prosthetic bypass (25, 48.1%) or aneurysmectomy and interposition graft or end-to-end anastomosis (27, 51.9%). At a mean follow-up of 18 months (SD ± 17.7), the primary, the primary assisted, and the secondary patency were 94.3%, 100%, and 100% respectively. No minor nor major amputations and no deaths were reported. **Conclusions:** In the endovascular era, our results highlight that regardless the specific characteristics—including age, comorbidities, and aneurysm anatomy—OR provides excellent early and mid-term outcomes with high patency and low complication rate compared with contemporary endovascular series reported in the literature.

## 1. Introduction

Popliteal artery aneurysms (PAAs) are rare vascular conditions that predominantly occur in patients in their sixth and seventh decades of life, with reported prevalence rates of 0.007% in males and 0.001% in females [1].

Despite their low incidence, PAAs are the most common aneurysms of the peripheral arteries; they are frequently bilateral (50%) and associated with aortic aneurysm in 30–40% of cases [1,2].

**Figure 1:**
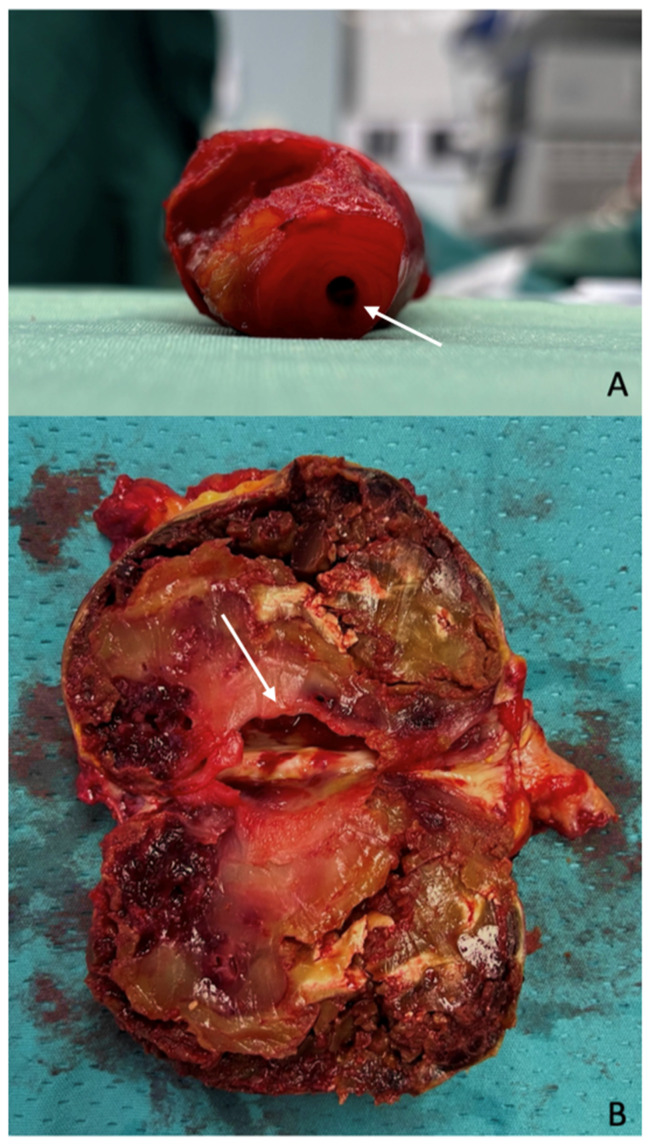
Thrombus within the aneurysm sac. A PAA following aneurysmectomy: (**A**) Residual arterial lumen within the thrombus. (**B**) The opening of the sac revealed a small residual lumen (arrows) surrounded by thrombus that potentially represents a source for distal embolization. Images taken from a case of PAA treated at our institution.

The most frequent (up to 21.2% of cases) and clinically significant presentation of PAAs is limb-threatening ischemia, resulting from recurrent embolic events caused by thrombus formation within the aneurysmal sac (Figure 1).

Deep vein thrombosis (DVT) due to compression of the adjacent popliteal vein (1.9% of cases), a palpable mass in the popliteal fossa (5.8% of cases), and more rarely (1.9% of cases) aneurysm rupture have also been reported as initial presenting symptoms [1,2].

Given the high risk of ischemic complications, identifying PAAs before the onset of acute or chronic embolization is crucial to prevent deterioration of the distal vessels’ runoff and preserve limb outcomes.

Beyond the clear indication for intervention in case of threatening limb ischemia, the Society of Vascular Surgery (SVS) indicates repair in asymptomatic, fit patients if the PAA diameter reaches ≥20 mm (grade 1B) [3]. For patients at high surgical risk, delaying repair until the diameter reaches 30 mm (grade 2C) has been advocated [3].

However, high-level evidence and randomized clinical trials or meta-analyses remain lacking to establish the superiority of open (OR) versus endovascular repair (ER) in the elective setting. Currently, OR is indicated (level 2C evidence) for asymptomatic patients with a life expectancy over five years and with a suitable autologous vein for revascularization, while ER appears appropriate for patients with a limited life expectancy [3].

Against this background, we conducted a single-center observational study analyzing outcomes in a cohort of patients treated with OR for PAAs. We aimed to investigate the safety and efficacy of traditional surgery irrespective of patient age, clinical conditions, and life expectancy and compare our results with those reported in contemporary endovascular series.

## 2. Materials and Methods

### 2.1. Population

This study was conducted in the Vascular Surgery Unit of the University Hospital “Agostino Gemelli” (Rome, Italy) in accordance with the STROBE guidelines (Supplementary Table S1).

All patients presenting with a PAA who underwent interventional management of a PAA during the last four years (1 January 2020–31 December 2024) were entered into a prospectively maintained electronic database that was then retrospectively reviewed. A prospective follow-up was also performed.

**Figure 2:**
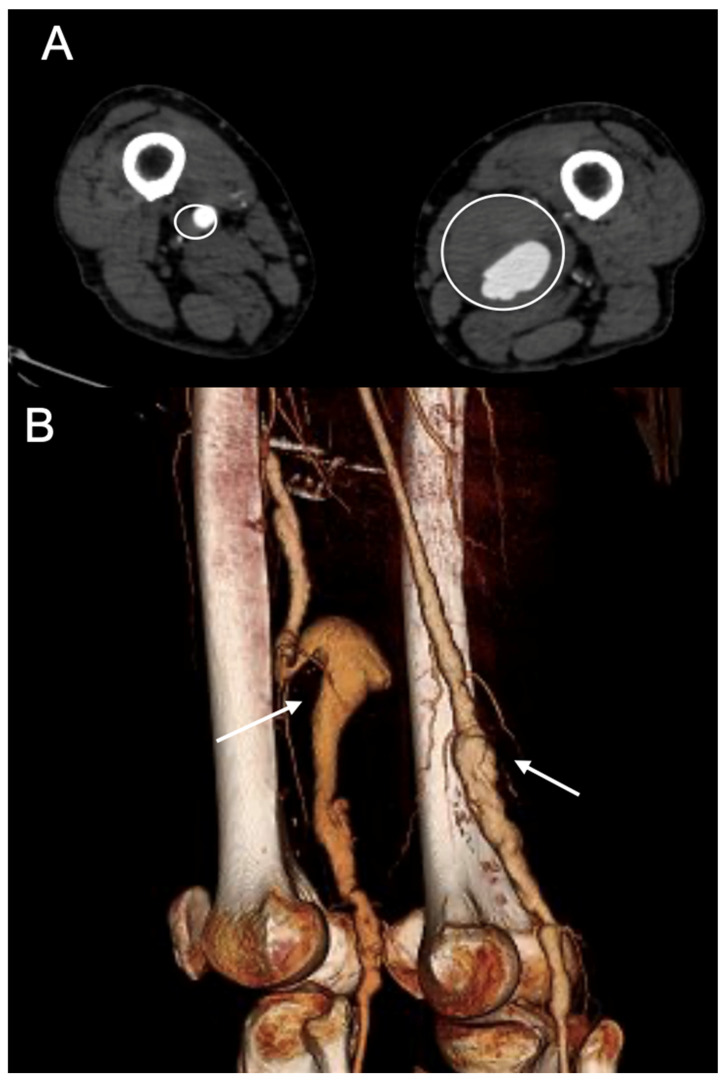
Preoperative computed tomography angiography of a patient presenting with a bilateral PAAA treated at our institution. (**A**) A case of bilateral popliteal artery aneurysm (PAA) treated at our institution. A transversal slice of the preoperative CTA (circles) and a 3D reconstruction (**B**) (arrows).

The inclusion criteria were as follows: (1) PAA > 20 mm in maximal diameter, (2) aged >18 years, (3) diagnosis confirmed by a contrast-enhanced computed tomography angiography (CTA), and (4) elective surgical treatment (Figure 2).

All symptomatic patients and those treated with an endovascular approach or emergently were excluded from the analysis.

### 2.2. Clinical Assessment and Preoperative and Postoperative Management

Clinical examination, Doppler ultrasound (DUS), and CTA were performed preoperatively to assess the aneurysm’s extent and size, the anatomical location of the aneurysm, and the patency of the runoff vessels. This comprehensive evaluation guided the selection of the most appropriate strategy and identified the most suitable distal target for bypass grafting.

Preoperative DUS was also performed in all cases to evaluate the quality of the great saphenous vein (GSV).

Our institution is a high-volume vascular center with extensive experience with both endovascular and surgical treatments. Indications for open or endovascular repair are discussed preoperatively by a multidisciplinary team (MDT) to balance each patient’s individual risk against the morphology and anatomy of the lesions. Although ER is preferred for patients with limited life expectancy, we consider as contraindication to the percutaneous approach an excessive size mismatch between proximal and distal sealing zones, major vessel tortuosity, popliteal aneurysms also extending to below-the-knee segments or presenting with multiple collateral branches originating from the sac, and clinical conditions contraindicating the use of dual antiplatelet therapy postoperatively (as in patients with a history of recent intracranial hemorrhage or hypersensitivity to the drugs or those at an increased risk of bleeding because of significant thrombocytopenia, large esophageal varices, end-stage renal disease on hemodialysis, or decompensated liver cirrhosis).

When open repair is indicated, we tailor the surgical approach based on anatomical considerations (PAA size and extent, femoropopliteal inflow, and quality of distal runoff). Techniques includes aneurysm exclusion through ligation and revascularization via an autologous or prosthetic bypass or aneurysmectomy in association with interposition graft or arterial end-to-end anastomosis.

Postoperatively, all patients received single antiplatelet therapy lifelong. All underwent clinical and arterial DUS evaluation with scheduled follow-up visits at 1, 3, 6, and 12 months postoperatively, followed by annual assessments.

Telephonic interviews were also regularly performed in between the scheduled clinical appointments to track the occurrence of any new symptoms, including calf or gluteal claudication or ischemic lesions of the foot.

### 2.3. Data of Interest

Data were collected from electronic medical records and telephone interviews.

All information were recorded anonymously in a digital database. The collected variables included the following: demographic (age, race, and sex), clinical data (medical history, symptoms, and presence of contralateral PAA, comorbidities, and cardiovascular risk factors), surgical and technical strategies used (surgical approach and technique, type of graft used, and need of additional procedures), postoperative complications and need for reintervention due to complications or loss of patency during in-hospital and follow-up periods, and mortality (in-hospital and during follow-up).

### 2.4. Endpoints

The primary endpoints were primary, primary assisted, and secondary patency. Secondary endpoints were amputation and early (within 30 days from the index procedure) and mid-term reintervention rate and overall mortality (in-hospital and at the last available follow-up).

Primary, primary assisted, and secondary patency rates were calculated using a Kaplan–Maier estimate based on the “first event” (arterial stenosis, occlusion, or reintervention) after the procedure.

### 2.5. Definitions

Primary patency was defined as uninterrupted graft function without any additional procedures to maintain or restore patency after the index surgery.

Primary assisted patency was defined as graft patency preserved through prophylactic or therapeutic interventions (e.g., surgical revision, endovascular angioplasty, or stenting) performed to prevent graft failure.

Secondary patency was defined as the restoration and continued function of the graft after occlusion, achieved through surgical or endovascular reintervention.

Amputation rate (AR) was defined as the proportion of patients requiring amputation due to revascularization failure measured from the time of the initial surgery to the last available follow-up.

### 2.6. Surgical Techniques and Strategies

As previously described, the surgical strategy was tailored based on anatomical and clinical considerations.

All procedures were performed by an expert consultant vascular surgeon under general anesthesia, except 3.8% (2) of interventions that were performed under locoregional anesthesia and sedation.

The choice of a posterior or medial approach depended on the aneurysm’s extent, the affected popliteal segment, and the surgeon’s preference.

**Figure 3:**
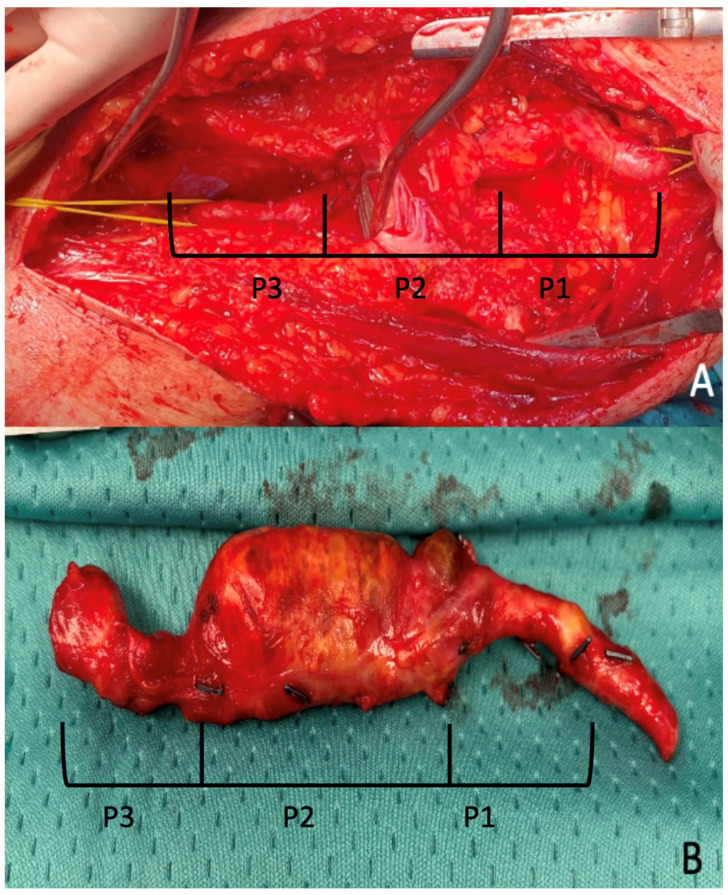
Intra-operative images of one case treated at our Institution. (**A**) Surgical access to a PAA affecting P1 portion of popliteal artery in a patient treated at our institution. (**B**) Surgical specimen following aneurysmectomy.

Interventions involved exclusion of the aneurysm either by ligation of the sac or by aneurysmectomy, followed by revascularization via a femoro-distal bypass, interposition graft, or end-to-end anastomosis (Figure 3).

Endarterectomy with or without additional patch angioplasty of the anastomosis site was performed whenever needed.

**Figure 4:**
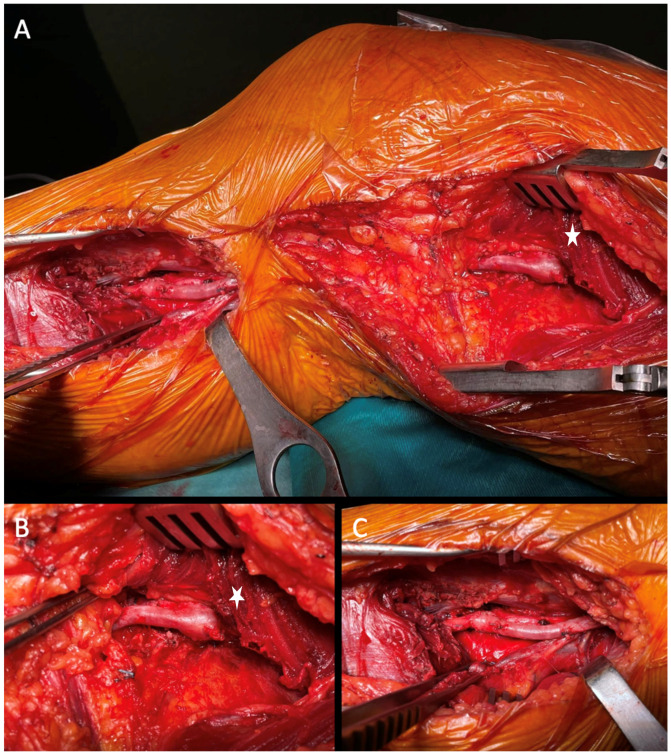
Intra-operative images of a femoropopliteal venous bypass. The picture illustrates a femoro-popliteal bypass in the inverted great saphenous (**A**) for treatment of a popliteal aneurysm via a medial approach. Details of the proximal ((**B**), star) and distal (**C**) anastomosis vein. Intraprocedural DUS was performed in all cases to check flow in the graft and distally before wound closure.

Whenever feasible, autologous grafts (great or small saphenous vein) were preferred over prosthesis (Figure 4).

### 2.7. Statistical Analysis

Continuous data are reported as median; categorical variables are reported as counts and percentages. Primary, primary assisted, and secondary patency rates were calculated with a Kaplan–Meier estimate based on the “first event” (arterial stenosis, occlusion, or reintervention) after the bypass procedure.

Means were compared using a *t*-test, and *p*-value < 0.05 was considered significant. Taking into consideration the small number of patients, no subgroups were analyzed.

Statistical analysis was performed with SPSS v. 25 statistics software (SPSS Inc., Chicago, IL, USA).

### 2.8. Ethics

This study was performed in accordance with the relevant guidelines. All data were anonymized before analysis to ensure participant confidentiality. All participants provided informed consent for the use and publication of their data and imaging.

As a retrospective service evaluation audit, approved by our Surgical Department and involving no prospective randomized study nor modification in patients’ care but application of already described treatment strategies, the formal approval of our Institutional Ethical Committee was not required.

## 3. Results

### 3.1. Clinical Data

**Figure 5:**
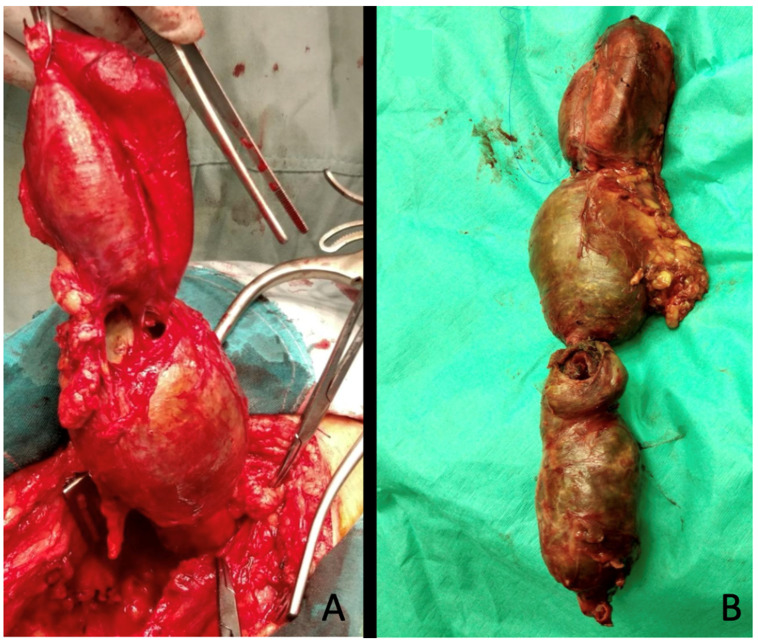
Intraoperative images of a large PAAA treated at our institution. (**A**) Intraoperative view showing the total excision of a PAA of 70 mm in maximal diameter. (**B**) Surgical specimen.

During the study period, 62 open interventions and 11 ER for PAAs were performed. According to the inclusion criteria, 49 (100% male) patients were included, accounting for 52 electively treated PAAs (median diameter 30.5 mm; range 20–75) (Figure 5).

The mean age of the population was 69.4 years (SD ± 9.0). The ASA (American Society of Anesthesiology) score was 3 in most cases (34, 69.4%). The main demographics and comorbidities are reported in Table 1.

Overall, 92.3% of PAAs (48 cases) were fusiform aneurysms, and the remaining (7.7%, 4 cases) had a saccular morphology. Runoff vessels were three in 17.3% of cases (9), two in of 71.2% cases (37), and one in 11.5% of cases (6).

Aneurysms involved the P1 segment in 38.5% of cases (20), the segment P2 in 48.1% of cases (25), and the P3 segment in 13.5% of cases (7).

### 3.2. Surgical Strategy

Aneurysmectomy and interposition graft was the most common approach (27, 51.9%), including one femoro-popliteal and 26 popliteo-popliteal grafts. Conversely, aneurysm exclusion via ligation was performed in 25 (48.1%) cases followed by femoro-popliteal (18, 34.6%), femoro-distal (3, 5.8%), and popliteo-popliteal (4, 7.7%) bypasses.

A prosthetic graft (polytetrafluoroethylene or Dacron) was used in 24 (46.2%) cases exclusively for above-the-knee reconstruction, while an autologous graft was selected in 27 (51.9%) (26 great saphenous and 1 small saphenous vein) ipsilateral in all cases. In one (1.9%) case a direct arterial end-to-end anastomosis was performed.

### 3.3. In-Hospital and Early Outcomes

A total of 19.2% (10) of patients required intensive care unit (ICU) admission for a single day due to their pre-existing comorbidities with a median stay of 0.2 days (range 0–1 day) (Table 2).

Early reinterventions were required in 3.8% of cases: in one case a patient who underwent aneurysmectomy and end-to-end popliteo-popliteal anastomosis for a P2 PAA needed reoperation using an autologous great saphenous vein graft on the first operative day due to bypass occlusion in the setting of an hypercoagulable state associated with SLE (systemic lupus erythematosus) disease and antiphospholipid antibody syndrome. Another patients was reoperated on postoperative day three following a femoro-tibial venous bypass for severe claudication: the DUS demonstrated a patent but demodulated bypass (peak systolic velocity 25 cm/s) caused by plication of the graft within the tunnel.

Minor perioperative complications occurred in three additional patients (5.8% of cases): one patient (1.9%) developed postoperative anemia requiring transfusion, and two patients (3.8%) developed DVT, which was managed with stocking compression and anticoagulation. No neurological complications involving the sciatic-popliteal or peroneal nerves were observed, and no wound issues were reported.

### 3.4. Follow-Up Data

**Figure 6:**
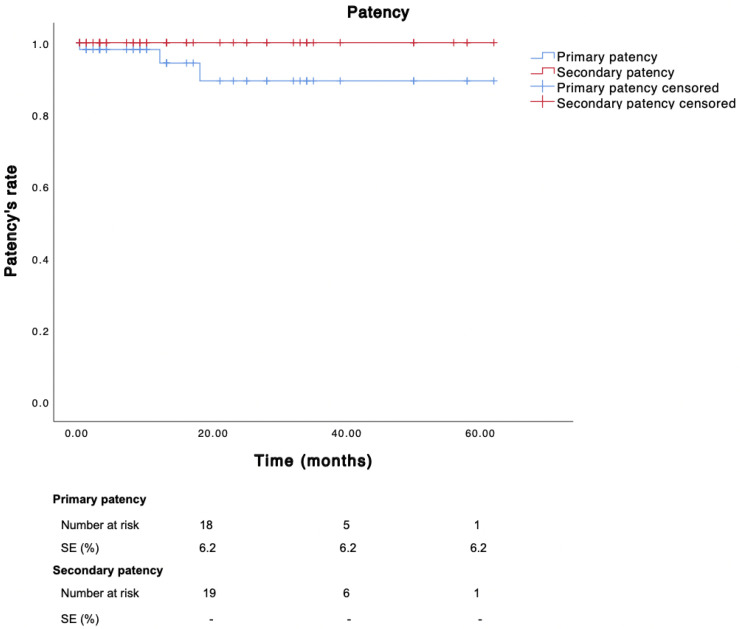
Kaplan–Meier estimate showing overall patency in the included population.

During a mean follow-up of 18 months (SD ± 17.7), the primary, primary assisted, and secondary patency rates were 94.3%, 100%, and 100%, respectively (Figure 6).

Overall, the reintervention rate was 7.7% (four cases): as previously detailed, two patients underwent revision of the bypass during the initial hospitalization, another patient required covered popliteal artery stenting due to bleeding from a collateral vessel of a saphenous graft 18 days after the initial procedure, and the remaining one underwent an urgent femoro-popliteal bypass 20 months after the initial procedure for an acute occlusion of a prior femoro-popliteal prosthetic graft. No major transfemoral amputations or minor amputation for pre-existing foot lesions were required.

## 4. Discussion

The primary goal of treating PAA is to exclude the aneurysm from the systemic circulation, preventing distal embolization or thrombotic occlusion and thus limb ischemia. According to current international guidelines, both endovascular and open strategies may be used based on clinical judgment and institutional expertise, vascular anatomy, and patient fitness [3].

Historically, open surgery has been the standard treatment. This may involve aneurysm ligation and bypass grafting or aneurysmectomy followed by interposition graft or end-to-end anastomosis. One of the main advantages of this approach is the high versatility allowing medial or posterior access tailored to each patient’s anatomy and the surgeon’s preference.

On the contrary, the more recent percutaneous repair, often favored in high-risk or frail patients, requires unique planning and procedural considerations.

Indeed, the popliteal artery endures substantial biomechanical stress from repetitive flexion of the knee and ankle. Therefore, before undertaking ER, one must assess size discrepancies between proximal and distal sealing zones, evaluate vessel tortuosity, and carefully analyze the burden of distal occlusive disease.

Specific anatomical features such as difficult access, involving below-the-knee segments or with several collateral vessels originating from the sac potentially leading to endoleaks and aneurysm growing, should discourage the use of ER [4].

Moreover, to date, no specific stent graft has been specifically designed for PAA management, potentially compromising procedural efficacy; the significant risk of graft thrombosis mandates the use of a loading dose of antiplatelet therapy before the intervention and to continue dual antiplatelet therapy (DAPT) for a minimum of 4–6 weeks postoperatively. This regimen may pose challenges in patients with contraindications to prolonged antiplatelet therapy or at increased risk of gastrointestinal bleeding.

Despite this limitation, several authors have advocated the superiority of ER when compared with OR, citing the lower wound complication rate and shorter length of hospital stay over open repair [3,4,5]. The minimal invasiveness of such an approach makes it potentially beneficial for high-surgical-risk patients such as older adult patients with multiple comorbidities and increased anesthesiologic risk [3,4,5]. Nevertheless, data comparing the results achieved with these two approaches are conflicting.

The current evidence indicates that in patients presenting with acute limb ischemia (ALI), open repair represents the optimal approach [5]. For instance, in a multi-institutional observational study analyzing the results achieved in symptomatic PAA, ER was associated with lower patency (30 days 70.4% vs 93%, and 1 year 47.6% vs 86.8%, *p* = 0.001) and higher amputation rates (30 days 14.8% vs 3.7%, *p* = 0.022, and 1 year 17.4% vs 6.8%, *p* = 0.098) compared with open surgery [6].

However, comparative results between ER versus OR in asymptomatic PAA remain inconclusive.

In this series we report satisfactory mid-term outcomes achieving high rates of primary, primary assisted, and secondary patency (94.3%, 100%, and 100%, respectively). Our results are in line with a previously published series on PAA OR.

To start with, in 2017, Joshi D. and colleagues [7] conducted a systematic review on elective treatment of PAA highlighting the scarcity of reported data. The one-year primary patency rate was 93.3% in the ER group versus 100% in the surgery group (RR 0.94, 95% CI 0.78 to 1.12; moderate-certainty evidence). The assisted one-year patency rate was similar in both groups (RR 1.00, 95% CI 0.88 to 1.13; moderate-certainty evidence). And no significant differences were observed at four years for either the primary or assisted patency rates (RR 1.00, 95% CI 0.76 to 1.32; moderate-certainty evidence).

On the other hand, the latest ESVS guidelines emphasize that the patient’s life expectancy should guide the choice of treatment for asymptomatic PAAs. Specifically, in those having an estimated life expectancy greater than five years, OR is recommended as the preferred approach, especially when a suitable autologous saphenous vein is available for bypass.

Indeed, more recent series demonstrates a clear long-term advantage for OR. For instance, in a retrospective multicenter analysis on 143 limbs by Ripepi et al. including patients treated for PAA in an elective setting, the primary patency at 1, 3, and 5 years was significantly higher in the OR group than in ER group (92.7%, 92.7%, and 81% for OR versus 77.3%, 67.9%, and 64.1% for ER, respectively, *p* = 0.01).

In our series, the overall freedom from reintervention was 92.3%, representing one of the main advantages of OR over ER. In this regard, Ripepi and colleagues demonstrated a higher rate of late occlusions in the endovascular group, which led to significantly lower freedom from reintervention at 1, 3, and 5 years in ER than OR (82.2%, 70.9%, and 70.9% for ER versus all of 95.7% for OR, *p* = 0.02) [8].

Accordingly, a retrospective analysis by Eslami et al. about 390 patients with asymptomatic PAAs (221 OR versus 169 ER) demonstrated superior outcomes in the surgical group: these patients experienced a significantly higher rate of major adverse limb event-free survival (95% vs 80%; *p* < 0.001, 100% in this series) and lower hazard of primary patency loss (HR, 0.25; 95% CI, 0.10–0.58; *p* < 0.05) during follow-up [9].

Finally, the study by Leake and colleagues also corroborates the hypothesis that OR outperforms ER in the long term: in their analysis encompassing 4880 popliteal artery aneurysm repairs (OR, 3915 versus ER, 1210), they reported that OR patients had lower odds of thrombotic complications (odds ratio 0.362 [0.155–0.848]; *p* < 0.001), few reinterventions (odds ratio, 0.275 [0.166–0.454]; *p* < 0.001), and better primary patency at 1 and 3 years (relative risk, 0.607 [*p* = 0.01] and 0.580 [*p* = 0.006], respectively) despite those subgroups having worse tibial runoff (odds ratio 1.949 (1.15–3.31); *p* = 0.013) than ER patients [5].

Our series describes the results achieved in a heterogenous group of PAA—regardless of preoperative clinical status, ASA class, comorbidities, and aneurysm anatomy—who experienced satisfactory outcomes and low morbidity rates. Although associated with a longer hospital stay, open surgery is more likely to be a definitive treatment with an acceptable rate of perioperative complications in this and prior studies (0.7% permanent nerve injury; 1.6% 30-day rates of MACEs; 2.7% procedure-related reinterventions; 0.0% vs 0.2% major amputation) [10,11,12].

Indeed, long-term follow-up data clearly indicate a lower patency rate for ER compared with OR and lower primary patency rates at 1, 5, 10, and 15 years (78%, 54%, 42%, and 31%, respectively) with a high rate of late complications (56.9%) and occlusion (37.4%) [13].

## 5. Conclusions

In the endovascular era, our results highlight that irrespective of patient characteristics such as age and comorbidities, OR guarantees excellent clinical results in both the early and mid-term, allowing for a high primary patency rate and low morbidities when compared with endovascular series reported in the literature.

## 6. Limitation

We acknowledge several limitations of the present study including the small number of patients, which restricts statistical analyses in terms of significance and weakens the strength of our conclusions. Additionally, there was no direct comparison group with endovascular repair or within the different open strategies used, preventing a robust assessment of relative outcomes.

## Figures and Tables

**Table 1:** Preoperative data.

**Preoperative Data**		
	*N* patients (total 49)	%
DEMOGRAPHICS		
Age (mean, SD)	69.4 SD +/− 9.0	
Male	49	100
Caucasian	49	100
COMORBIDITIES AND CARDIOVASCULAR RISK FACTORS		
Dyslipidemia	37	75.5
Hypertension	41	83.7
Tobacco use	25	51.0
Diabetes type II	12	24.5
CKD	1	2.0
AAA	12	24.5
Contralateral PAA	22	44.9
ASA CLASS		
1	-	-
2	10	20.4
3	34	69.4
4	5	10.2
	*N* cases (52)	%
CLINICAL PRESENTATION		
Asymptomatic	37	71.2
Chronic limb ischemia	11	21.2
Deep vein thrombosis	1	1.9
Palpable mass	3	5.7
ANATOMY OF THE ANEURYSM		
Fusiform	48	92.3
Sacciform	4	7.7
Diameter (median)	30.3 (range 16–75)	
Bilateral	25	48.1
SITE		
P1	20	38.4
P2	25	48.1
P3	7	13.5
RUNOFF VESSELS		
One	6	11.5
Two	37	71.2
Three	9	17.3

SD—standard deviation; ASA—American Society Anesthesiology; CKD—chronic kidney disease; AAA—abdominal aortic aneurysm; PAA—popliteal artery aneurysm.

**Table 2:** Surgical and postoperative data.

SURGICAL DATA		
	*N* cases (total 52)	%
TYPE OF VASCULAR RECONSTRUCTION		
Aneurysm ligation and bypass	25	48.1
Aneurysmectomy and interposition graft/end-to-end anastomosis	27	51.9
APPROACH		
Posterior	20	38.5
Medial	32	61.5
GRAFT		
Autologous vein	27	51.9
Prosthesis	24	46.2
IN-HOSPITAL DATA		
ICU stay	Median 0.2 days (range 0–1)	
Hospital stay	Median 4.0 days (range 2–8)	
Complications	5	9.6
Deep vein thrombosis	2	3.8
Anemia	1	1.9
Occlusion of the revascularization	2	3.8
FOLLOW-UP DATA		
Follow-up length	Mean 18 months (SD ± 17.7)	
Reintervention	4	7.7
Amputation (minor/major)	-	-
Overall mortality	-	-
Primary patency	49	94.2
Primary assisted patency	52	100
Secondary patency	52	100

ICU—intensive care unit; FU—follow-up.

## Data Availability

The raw data supporting the conclusions of this article will be made available by the authors on request.

## Supplementary Materials

The following supporting information can be downloaded at https://www.mdpi.com/article/10.3390/diagnostics15202608/s1: Table S1: STROBE checklist.

## Abbreviations

The following abbreviations are used in this manuscript:

ICU	Intensive care unit
FU	Follow-up
SD	Standard deviation
ASA	American Society of Anesthesiology
CKD	Chronic kidney disease
AAA	Abdominal aortic aneurysm
AR	Amputation rate
DUS	Doppler ultrasound
DVT	Deep vein thrombosis
CTA	Computed tomography angiography
PAAs	Popliteal artery aneurysms
OR	Open repair
ER	Endovascular repair
